# A correlation between seasonally changing photoperiod, whole body lipid, and condition factor in juvenile spring Chinook salmon (*Oncorhynchus tshawytscha*)

**DOI:** 10.1371/journal.pone.0285380

**Published:** 2023-05-18

**Authors:** Dina K. Spangenberg, Abby E. Fuhrman, Donald A. Larsen, Brian R. Beckman

**Affiliations:** Environmental Fisheries Science Division, Northwest Fisheries Science Center, National Marine Fisheries Service, National Oceanic and Atmospheric Administration, Seattle, Washington, United States of America; National Cheng Kung University, TAIWAN

## Abstract

The regulation of lipid stores is a central process for the physiology and ecology of fishes. Seasonal variation in lipid stores has been directly linked to survival of fishes across periods of food deprivation. We assessed whether a seasonally changing photoperiod was correlated to seasonal changes in energetic status to help better understand these important processes. Groups of first feeding Chinook salmon fry were introduced to a seasonal photoperiod cycle, but the point of entrance into the seasonal cycle varied from near the winter solstice (December), to either side of the spring equinox (February & May). Temperature and feeding rate were similar for all treatments. Subsequently, condition factor and whole body lipid content were assessed through a seasonal progression. Throughout most of the experiment, length and weight did not differ between the different photoperiod treatments, however whole body lipid and Fulton’s condition factor did. Furthermore, changes in both whole body lipid and Fulton’s condition factor in all treatment groups followed a similar seasonal pattern that was inversely related to day length (highest K and lipid levels found during days with the least light). These results suggest that regardless of age or size, there is a correlation between seasonal changes in photoperiod and changes in body composition in juvenile Chinook salmonids.

## Introduction

Seasonal changes in whole body lipid level (hereafter lipid levels) and Fulton’s condition factor (hereafter *K*) are common in naturally rearing juvenile salmonids. In general, lipid levels are relatively high in the late summer/autumn, decline in the winter, and then increase in the spring [1–5]. A similar pattern of lipid accumulation and depletion has been observed in a variety of other fish species [6, 7]. While the exact timing and magnitude of these changes in energy reserves may differ from year to year and/or between species, this similar pattern of seasonal change in energetic status suggests that there are common drivers influencing this physiological process.

Variation in the accumulation of energy reserves in the autumn, or variation in the depletion of energy reserves throughout the winter, is correlated with over-winter survival of juvenile freshwater fishes [8] including salmonids [9, 10]. As fish enter winter, feeding is reduced and survival relies on energy reserves exceeding the cumulative metabolic demand over the winter [8, 11]. Many studies examining this seasonal pattern of energy depletion have focused on environmental factors such as food availability and water temperature that dictate the reduction in energy reserves [12, 13]. In addition, behavioral trade-offs between foraging and predation risk have been examined in studies relating appetite changes to seasonal depletion of energetic stores after the autumn equinox. Both modeling exercises and empirical data suggest that reduced appetite during the winter is related to the degree of lipid depletion [14, 15].

Other organisms experience similar seasonal changes in lipid levels as do fish. Some birds have clear seasonal patterns of lipid deposition [16, 17]. Furthermore, birds have been shown to have specific behavioral and physiological mechanisms that result in seasonal patterns of fattening prior to seasonal energetic stresses, such as long-term migration, that result in energy depletion [18, 19]. Similarly, mammals that typically hibernate develop large fat stores through seasonal changes in appetite and physiology prior to initiating hibernation in the late autumn or winter [20].

Recent studies suggest similar mechanisms may be operative in fish during the summer/autumn and result in specific increases in energy reserves during this period. Giacommi and Shuter [21] suggested a physiological trade-off of energy allocation, from growth in bone and muscle tissue to an accretion of lipid reserves, might be responsible for increases in lipid in the autumn in fishes. Martin et al. [22] reviewed patterns of energy storage and use in fish and came to a similar conclusion, that there is an increase in lipid storage in the autumn. Thus, a basic physiological rhythm may underlie seasonal patterns of change in energy reserves in temperate juvenile fishes: seasonal fattening centered on the autumnal equinox followed by seasonal reduction in energy reserves due to metabolic demands exceeding consumption during the winter. Understanding the physiological mechanisms and environmental drivers that underlie seasonal patterns of energy change in juvenile fishes might allow us to better understand current ecological processes leading to variation in over-winter survival.

This experiment was designed to isolate the effect of a seasonally varying photoperiod from the effects of size and age on lipid levels and *K* (a proxy for lipid) in juvenile spring Chinook salmon (*Oncorhynchus tshawytscha)*. Treatment groups were created by introducing first-feeding fry of the same age and size into different tanks with artificial lighting mimicking the natural photoperiod cycle at differing seasonal periods: winter solstice (December), early spring (February) and late spring (May). Since temperature was held constant and feeding rate was paired between treatments, we could directly correlate physiological status to photoperiod. We hypothesized that changes in lipid level and *K* would be correlated with seasonal changes in photoperiod and not with either age or size.

## Materials and methods

### Fish and rearing

On 17 November 2006 approximately 10,000 eyed embryos from 13 families of hatchery origin spring Chinook salmon were transferred from the Yakima River spring Chinook Salmon Supplementation and Research Facility (Cle Elum, WA, rkm 297 on the Yakima River) to the Northwest Fisheries Science Center (Seattle, WA) for experimental rearing. Family groups were randomly placed into 2 different Heath trays (Marisource, Fife, WA) and incubated under 24-hour darkness, with recirculating dechlorinated municipal water at 10°C. Embryos were monitored daily to remove mortalities and to determine developmental stage. When there was no visible yolk sac and the ventral body wall was nearly or completely fused, all fry were removed from incubation trays in March 2007 and randomly placed into experimental tanks (ponded) and first exposed to light. Each circular fiberglass experimental tank (1.4-meter diameter) was tented with dark plastic to isolate it from any external light and equipped with both an individual light source (60 W incandescent light bulb) and timer for photoperiod manipulation. Freshwater was sourced from a closed water recirculation system with biofiltration, ozonation, and ultraviolet sterilization and temperatures averaged 10°C (+/- 1°C). Water depth in tanks was 0.5m. Fish were fed Bio-Oregon (Warrenton, OR) fish feed with the following nutritional content; protein 47.0%, fat 18.0%, ash 12.0%, and moisture 8.5% throughout the study.

This study was conducted at the Northwest Fisheries Science Center (NWFSC), Seattle in accordance with University of Washington Institutional Animal Care and Use Committee (IACUC) protocol number 2313–90.

### Experimental design

**Fig 1:**
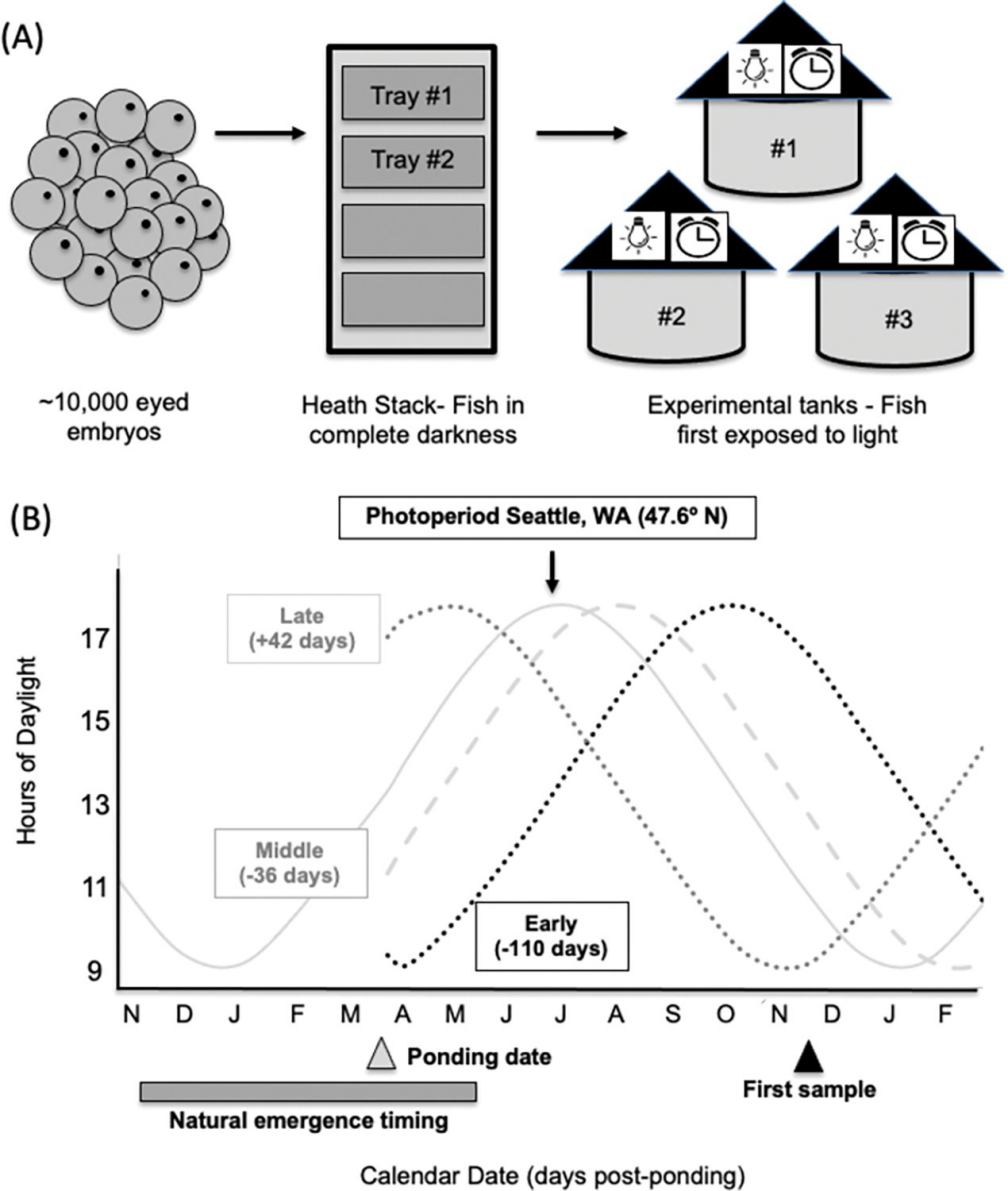
(A) Graphic representation of experimental design illustrating different environments (incubation and experimental tanks) to which eyed embryos and groups of fish were exposed. (B) Experimental timeline, milestones, and treatment (photoperiod) cycles (early = black dots, middle = grey shaded, late = grey line). Grey triangle indicates the date (20 March 2007) fish were transferred ("ponded") from completely dark egg incubation trays into respective experimental photoperiod treatments (early, middle, late). Black triangle indicates the date (19 November 2007) when fish were first sampled for physiological measures.

On 20 March 2007, fry from 2 Heath trays were removed and evenly mixed into 3 different tanks (300 fish per tank; 900 fry total) with different light/dark schedules (photoperiods). Fry were moved from complete darkness and first exposed to light upon transfer to rearing tanks, which is a common practice in salmon hatcheries in the Pacific Northwest (Piper et al. 1982) [23]. All fish were ponded on the same calendar day and were the same age; photoperiods in the tanks at ponding were different and corresponded to photoperiods found on 1 December, 15 February, or 1 May (Fig 1). These photoperiod cycles were selected as they represent early, middle, and late dates for when fry emerge into the water column from incubation gravels and begin feeding along the spectrum of natural emergence dates for Chinook salmon throughout the Pacific Northwest (North American). Photoperiod was adjusted weekly for each tank to account for seasonal changes, as found in Seattle, Washington (Lat 47° N). Batch weights from each tank (three batches of 20–50 fish per batch) were conducted monthly to assess average size and subsequently calculate a feeding rate (g feed/tank). To control for potential appetite differences due to variation in photoperiod, all groups were pair-fed, meaning that the ration for all tanks was determined based on the tank with the lowest satiation feeding rate. Pair-fed rates for a given month were determined as follows: on a given day fish in all tanks were fed by hand to satiation (based on visual assessment of when all fish in the tank quit feeding) and the amount of feed distributed to each tank was determined. Feeding rate (g feed/g fish) was determined for each tank based on total feed distributed/total weight of fish in the tank. For the succeeding month, all tanks were fed at a rate corresponding to the one tank with the lowest satiation feeding rate. This feeding rate varied from 2.3–1.2% body weight/day (feeding rate diminished as the fish increased in size).

The pair feeding approach was used to limit variation in growth related to potential variation in appetite between groups. Our goal was to keep fish size equivalent as there are well-known relationship between fish size and both lipid content and *K* (*K* and lipid increases with size). It’s also well known that fish appetite changes seasonally [24]. By utilizing a pair feeding approach we attempted to isolate seasonal changes in physiology from seasonal changes in appetite and related size variation.

### Sampling

At the time of ponding, 30 fish (15 from each Heath tray) were measured for fork lengths (mm), weight (gm) and *K (hereafter FL & W)*. After ponding, fish were sampled 5 additional times throughout the investigation with the next sample occurring on November 19. Subsequent samples occurred at a frequency ranging from 6–11 weeks. On each sampling date 25 fish were randomly selected from each treatment tank, anesthetized with a lethal dose (500mg l^-1^) of buffered tricaine methanesulfonate solution (MS-222, Argent Chemical Laboratories, Redmond, WA) and measured for fork length and body weight. Carcasses were individually tagged and stored in plastic bags at -20° C for later processing.

### Laboratory analysis

Percent whole body lipid levels were measured via a two-step process. Fish were first processed to obtain wet and dry mass weights, and then dried fish were processed for lipid extraction. Frozen fish were cut into small (~0.5 cm^3^) pieces, placed into pre-weighed aluminum pans, weighed for wet mass and then dried in an oven at 105°C to a consistent weight. After approximately 48 hours, fish were removed from the oven and weighed for dried mass. Whole body percent moisture (moisture) was determined by the difference between the wet and dry mass. Dried fish pieces were homogenized in an electric coffee grinder to create a fine powder and this was stored in an airtight scintillation vial in a desiccator cabinet until time of lipid extraction. From the dried fish samples, lipid levels were determined by the gravimetric method of the Association of Official Analytical Chemists [25] wherein methylene chloride served as a solvent for lipid extraction.

### Data analysis-approach

In this study we randomly distributed fish of the same age and size into differing seasonal photoperiod cycles and then monitored fish characters across 9 months. We examined these results within a context of changing seasonal photoperiod by fish metrics (FL, W, K) to relative photoperiod, as indexed by photoperiod date when the fish were sampled. This analysis encompassed all samples from all tanks of fish into a single assessment. A significant correlation between physiological state and photoperiod day allowed us to make the inference that physiological state was correlated with changing photoperiod. We describe and discuss this approach as correlational–examining fish metrics in relation to photoperiod date across the whole experiment. In addition, we tested for differences in mean fish metrics between tanks on given sampling dates to assess whether we had successfully controlled for potential differences in fish growth between treatments with our pair feeding approach. Significant differences in fish size at a given date would suggest that the pair feeding approach had failed. We applied an orthogonal approach–a comparison of mean fish metrics between tanks of fish on a given sampling date. We will include a complete orthogonal analysis of all fish metrics as they represent a traditional data analysis. However, we note that our treatments (photoperiod) were not replicated by tank. So, all results of the orthogonal analysis were simply assessed as a difference between tanks of fish and not a treatment effects.

### Data analysis-methods

Factorial ANOVAs were used in the orthogonal approach. FL, W, and *K* data were log transformed to normalize distributions and three individual fish were identified as outliers and excluded from all factorial ANOVAs as they contained values that were far outside the normal range of size or lipid levels (> 2 standard deviations from the mean). *K* was calculated based on Fulton’s equation and scaled for units of measurements: *K* = (W/FL^3^) x 100,000, where W = weight (g) and FL = fork length (mm).

Simple regression was used in the correlation assessment of fish size with photoperiod date. Seasonal changes in lipid levels and *K* were described using polynomial regression. The relationships between lipid levels, FL, W, and *K* were also examined by simple linear regression. Data were analyzed using R software, version 3.3.3 (R Foundation for Statistical Computing, Vienna, Austria. https://www.R-project.org/). Statistical significance was set at a level of α = 0.05.

## Results

### Size, *K*, and lipid level by age

**Fig 2:**
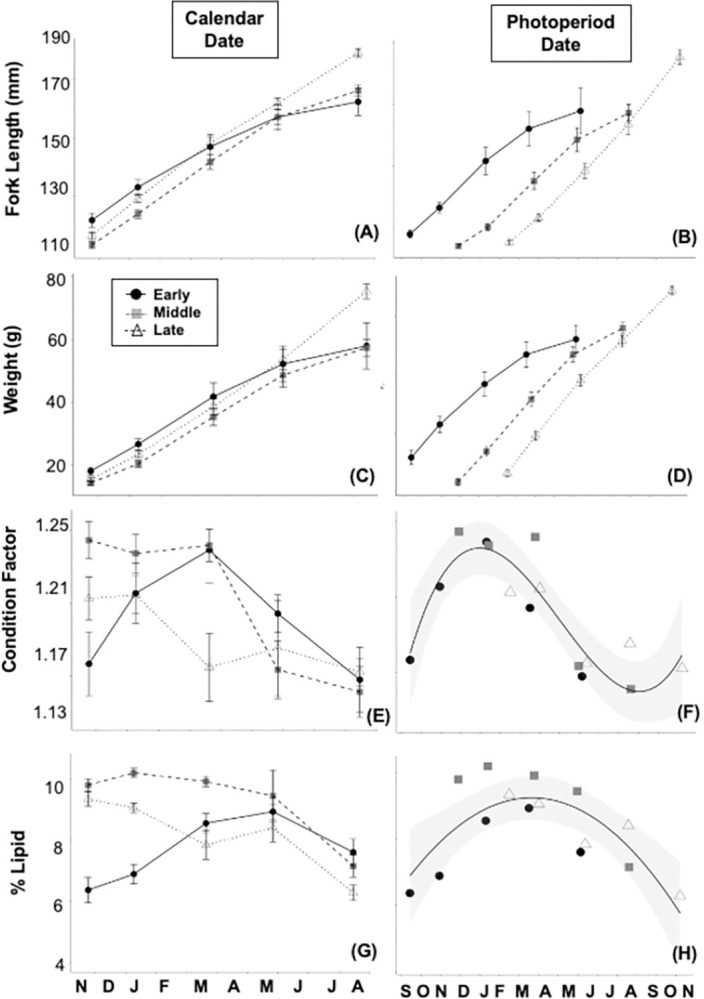
Mean (± SE) length (A, B), weight (C, D), condition factor (E, F) and percent lipid (G, H) aligned by calendar date (A, C, E, G) and by photoperiod date (B, D, F, H) of Yakima spring Chinook salmon reared under three different photoperiod regimes, early emerge (circles), middle emerge (squares), and late emerge (triangles). Best fit polynomial regression curves (condition factor: y = 0.1386 + 0.0076x + 0.000017x^2^ + 1E-08x^3^, *p* = 0.00039, *r*^2^ = 0.798; % lipid: y = -3.483 + 0.05869x + -0.00006778x^2^, *p* = 0.00258, *r*^2^ = 0.630) are added to photoperiod date graphs to show seasonal trends in condition factor and lipid profiles.

At the time of ponding fish were sourced from the same population and were evenly distributed between the 3 tanks. The mean FL, W, and *K* for fish in all tanks was 34.1 ± 0.74 mm (mean ± SD), 0.31 ± 0.03g, and 0.77 ± 0.09 respectively and the cv was found to be low. Fish grew steadily throughout the study and final mean lengths and weights by tank ranged from approximately 165–180 mm and from 55–75 g in July, 16 months post-ponding (Fig 2). We found significant effects of tank, date and tank by date for length while there were significant effects of tank and tank by date for weight (Table 1). In general, fish were of similar size across the 1^st^ 4 sampling dates but size diverged over the last growth stanza and fish from the late photoperiod were bigger than fish from the other photoperiod treatments on the final sampling date. The source of this divergence is unclear, but it could be a result of a miscalculation of pair feeding rates on the last stanza or sampling error on the last date.

**Table 1:** Results of ANOVA analysis of tank means of length, weight, *K* and percent lipid through time for juvenile spring Chinook salmon reared.

		*F*-value	*p*-value
**Length**			
	tank	7.5	<0.001
	date	39.9	<0.001
	tank x date	3.2	0.003
**Weight**			
	tank	4.4	0.013
	date	104	<0.001
	tank x date	2	0.049
**Condition**			
	tank	2.2	0.110
	date	7.2	0.004
	tank x date	2.8	0.005
**Lipid**			
	tank	24.5	<0.001
	date	12.9	<0.001
	tank x date	8.2	<0.001

In contrast, fish from different tanks differed significantly for both *K* and lipid at the first sampling date with further differences found on the 3^rd^ sampling for *K* and the 2^nd^ and 3^rd^ sampling for lipid (Fig 2). Note, that the 1^st^ sampling event occurred 243 days after photoperiod treatments had been initiated. At the final sampling date both *K* and lipid levels had converged to similar levels for all three groups. These patterns are reflected in the overall ANOVA results with significant effects for tank, date and tank by date for both *K* and lipid level (Table 1).

### Size, *K*, and lipid level by photoperiod

**Fig 3:**
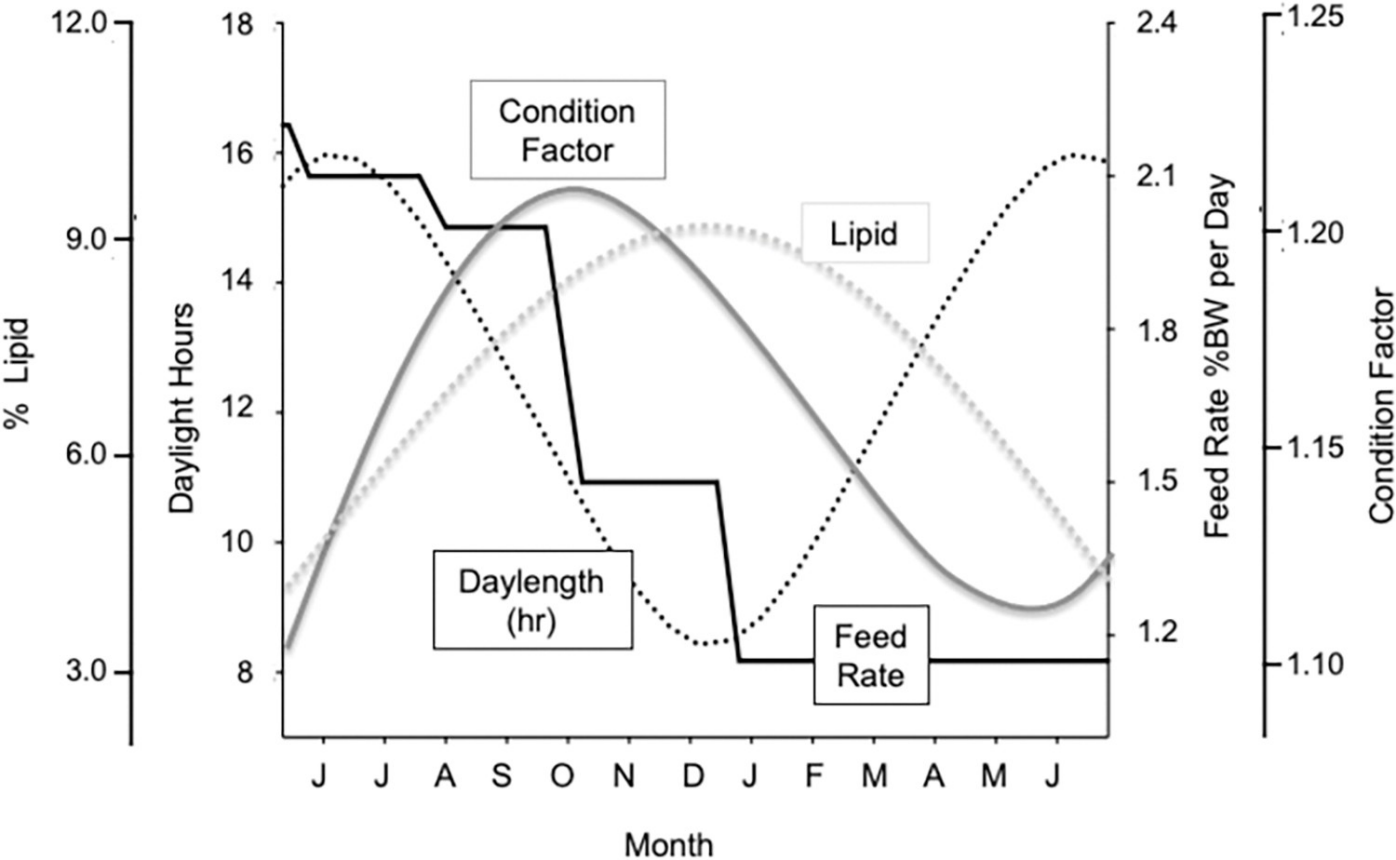
Graphic representation of polynomials for *K* (gray solid line) and percent lipid (gray dotted line) plotted with hours of daylight (black dotted line) and feed rate (black solid line).

When transitioning from the orthogonal to the correlational analysis, length and weight profiles became parallel to each other rather than over-lapping (Fig 2). More interestingly, patterns of change in *K* and lipid converged on the photoperiod axis, with 2^nd^ or 3^rd^ order polynomials reflecting much of the variation in each measure. For all tanks, maximum *K* and lipid levels were found during photoperiod intervals encompassing the winter solstice through the spring equinox (Dec–March) with both *K* and lipid level declining subsequent to photoperiod increases in the spring (Fig 3).

### Correlations among size, *K*, lipid, and photoperiod

**Fig 4:**
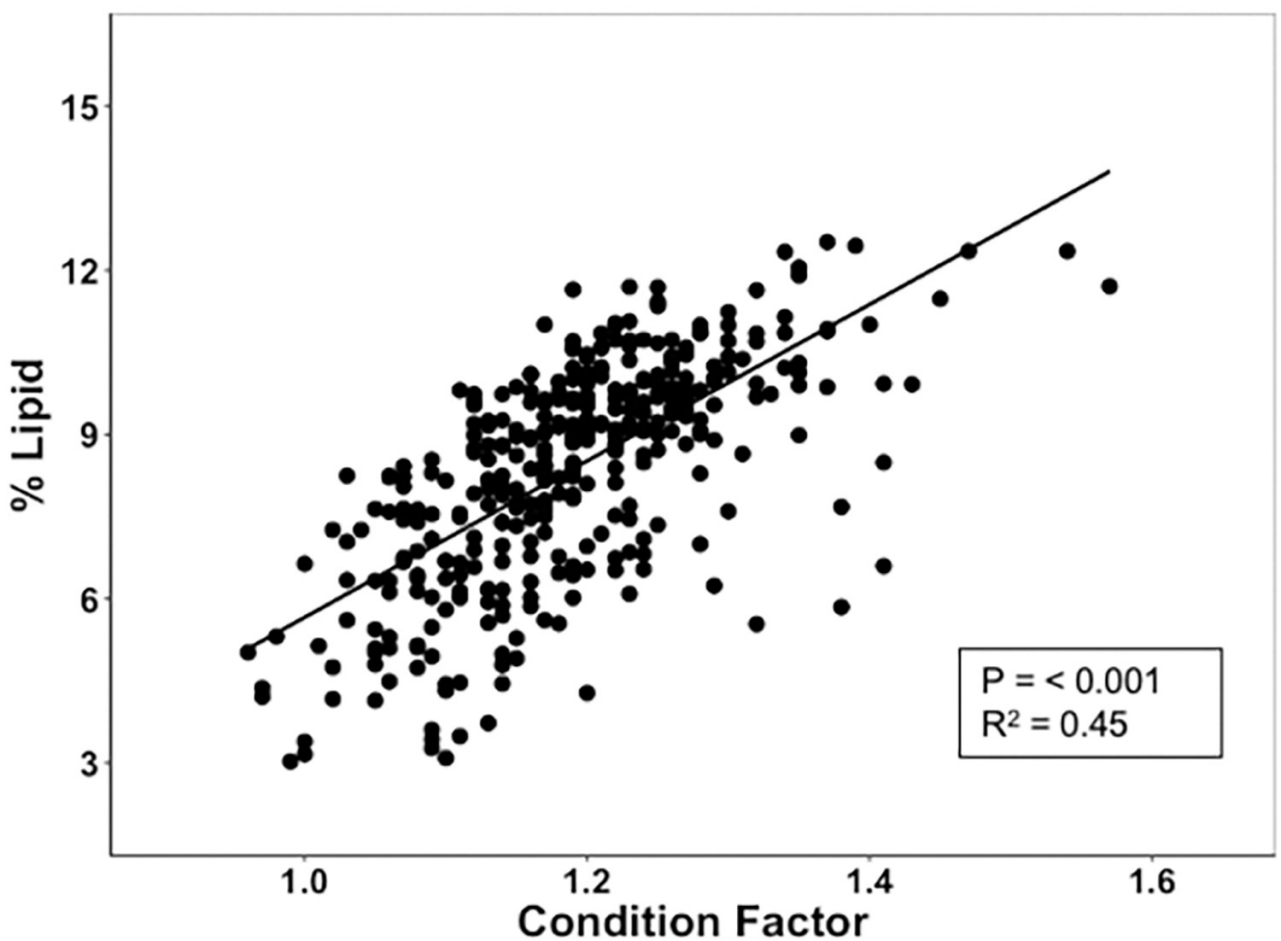
Linear regression of *K* vs. percent lipid for individual Yakima spring Chinook salmon.

The experimental design resulted in a decoupling of size from *K* and lipid. No significant relationship was found between weight and lipid levels (*P* = 0.127) and correlations between fork length and lipid level were poor (P < 0.001, r^2^ = 0.03) (Table 2). In contrast, *K* was well correlated with lipid level, with a useful portion of the variation in lipid included (P = < 0.001, r^2^ = 0.45) (Fig 4).

**Table 2:** Correlations among length, weight, *K* and percent body lipid.

	Lipid	
	r^2^	p
Length	0.03	< 0.001
Weight		0.127
Condition	0.45	< 0.001

## Discussion

Many different designs have been used for studies on the effects of photoperiod on physiology and development of fishes [26, 27]. Some experiments feature abrupt change in natural light schedules while others utilize “skeleton” photoperiods that incorporate large step-changes in constant daily photoperiods (i.e. 8:16 light:dark to 16:8 light:dark). In this experiment the identical photoperiod schedule (weekly change in minutes of light/day) was used for all treatments. Thus, any differences among treatments were generated by introducing post-emergent fry into the same natural photoperiod schedule but at different timepoints (Dec 1, February 15, May 1) during their natural period of emergence (Nov-May). In addition, a pair-feeding design, where ration was set to the group with the lowest appetite during a given period of the experiment, was used to control for potential differences in appetite among treatments. The intent of this experimental design was to have fish of the same age and size in different seasonal phases of the same photoperiod cycle at the same experimental time, effectively isolating the photoperiod effect from size or age (see Beckman et al. [28]). This approach allowed us to infer that the experimental differences in lipid levels and *K* were correlated with photoperiod change across all groups. Many studies have demonstrated seasonal changes in energy stores [1, 4, 12, 13]. However, the novelty of our experiment is that fish were held at a constant temperature and equal feeding rates, effectively isolating photoperiod from the normal seasonal changes in temperature and feeding, thus generating some novel insight as changes in temperature and feeding rate are often assumed to be the primary drivers for seasonal changes in lipid and *K*.

### Seasonal patterns

**Fig 5:**
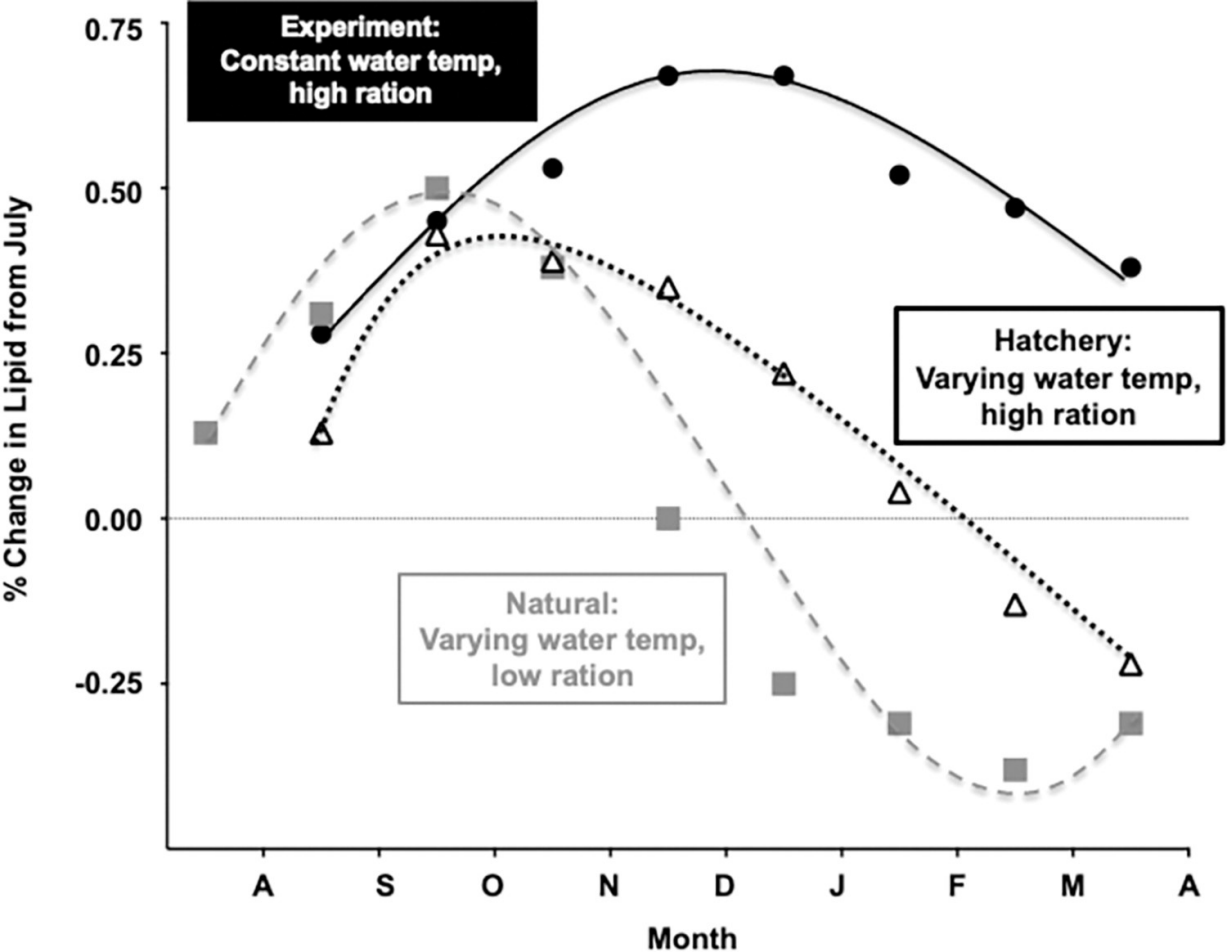
Comparison of seasonal changes in whole body lipid for juvenile Yakima River spring Chinook salmon either rearing naturally in the Yakima River (Beckman et al. 2000, squares), rearing in the Cle Elum hatchery under standard *K*s, with seasonally changing temperature and feeding rates (Larsen et al. 2006, triangles) or this study (circles). There is a common increase in lipid from the summer solstice through the fall, however the magnitude of change in lipid levels vary according to feeding rates and metabolic demand characteristics of the different environments.

Previous studies in juvenile Chinook salmon suggest that our results mimic a natural phenomenon and are not a laboratory artifact. Beckman et al. [2] examined the seasonal physiology of naturally rearing juvenile spring Chinook salmon in the Yakima River and Larsen et al. [5] assessed seasonal changes in lipid levels in both hatchery and naturally rearing juvenile spring Chinook salmon in the Yakima River. Both studies describe a pattern of seasonal increase in lipid levels during the autumn and a decrease in the winter, similar to what we found in this study (Fig 5). However, because these experiments occurred in different environments (natural vs hatchery) under different experimental conditions (feeding rates, diets, and temperatures), the absolute lipid levels and the timing and magnitude of this lipid decrease varied. These comparisons, among the same stock of fish reared in a laboratory, a production-scale hatchery, and a river provide further evidence supporting a general pattern of lipid accretion in the autumn that is correlated with a seasonal decrease in photoperiod.

For fish in general, energy reserves typically fluctuate in correspondence to seasonal changes in the external environment. In the spring and summer, when temperatures and food supply are favorable, fish grow in both length and weight, however in the late summer and fall when temperatures, day length, and food supplies are declining, fish may physiologically shift from growth in length to just growth in weight and increase relative lipid levels [7, 22, 29, 30]. Thus, it appears that seasonal variation in lipid reserves is common among many fish species and may result from a common physiological process.

### Comparative physiology: Seasonal increases in lipid reserves

Seasonal increases in lipid reserves are common in many vertebrates and have been well-studied with regard to pre-hibernation and pre-migratory fattening in mammals and birds [17, 20, 31, 32]. Photoperiod has been found to play an important role in the increase in fattening related to both of these physiological processes [33, 34]. In addition, neural and endocrine mechanisms modulating the seasonal fattening process have been identified [35]. Taken together, our results showing that lipid levels increase in the fall coinciding with a decreasing photoperiod corroborate previous findings within the context of comparative vertebrate physiology.

A common paradigm for the role of autumnal fattening is that this process provides energy stores for the winter when metabolic demands exceed food availability [36, 37]. The validity of this paradigm for juvenile fish is supported by a number of studies that have found significant differential over-wintering mortality in relation to relative lipid level [9, 21, 38, 39]. There appears to be a common ecological driver for this pattern: greater lipid levels prevent starvation and death in the winter due to the exhaustion of energetic reserves needed to maintain metabolism. Thus, one might conclude that selection favors physiological processes that optimize autumnal fattening so as to reduce winter mortality due to exhaustion of energy reserves.

### Lipid level and *K*

*K* measures are often found in reports on the ecology of naturally rearing fish. Length and weight are easily measured and *K* results from a simple calculation [40–42]. In general, fish with a lower *K* are considered to be leaner with lower energy reserves than fish with a higher *K*. Some researchers have documented a positive relationship between *K* and lipid levels [43–46] while other studies have found weak relationships between Fulton’s *K* and percent whole body lipid or energy density [47, 48].

In many studies lipid level is directly related to size [49]. In the current investigation we found little relationship between size and either lipid level or *K* when results from all dates were combined. Thus, the photoperiod treatment resulted in a decoupling of size and lipid level, allowing us to then examine the relationship of *K* to lipid without size co-varying with both. Subsequently, we found a significant correlation between *K* and lipid level. This relationship occurred over a range of ages, sizes, and seasons suggesting that it is relatively robust for use in studies of juvenile Chinook salmon of relatively similar sizes. *K* is not a direct measure of lipid level but large variation in *K* of fish of similar ages and sizes should provide a reasonable inference that lipid levels vary between those individuals (or populations).

### Implications

Evolutionary processes have driven animals to use proximate environmental cues to develop seasonal patterns of energy storage [11, 21]. These patterns of energy storage can be related to seasonal patterns of food availability and energetic costs that may allow juvenile fish to better survive periods of energy depletion during the winter. The survival advantages provided by seasonal patterns of energy accumulation guided by proximate environmental cues may be diminished as environmental conditions (food availability, temperature/metabolic costs) change with changing climate and become disconnected with current proximal environmental cues. Thus, it is important to understand the physiological mechanisms driving patterns of energy accumulation in order to predict the responses of fish as the relationships between environments, and the proximate cues relating to environment change [11, 50]. As photoperiod does not vary with other environmental conditions, at least one of the major influences driving the seasonal accumulation of energy will stay constant. However, the role photoperiod plays in guiding seasonal energy accumulation may diminish as seasonal patterns of temperature and food availability diverge from seasonal photoperiods with the onset of climate change.
